# Excessive Daytime Sleepiness in Patients With Hypertension: A Systematic Review

**DOI:** 10.7759/cureus.50716

**Published:** 2023-12-18

**Authors:** Yazeed Almansouri, Abdulrahman Alsuwatt, Mazen Alzahrani, Muteb S Alsuwat, Riyadh Alamrai, Wasaif S Alsuwat, Bader H Almansouri, Abdulkareem F Al Bahis

**Affiliations:** 1 Medicine, Taif University, Taif, SAU; 2 Family Medicine, Security Forces Hospital, Riyadh, SAU; 3 Family Medicine, Ministry of Health, Khamis Mushait, SAU

**Keywords:** sleep apnea and hypertension, obstructive sleep apnea (osa), hypertensive patients, hypertension, excessive daytime sleepiness

## Abstract

We must remember that there are many subclinical cases of obstructive sleep apnea (OSA), even if the patient or family members typically become aware of it through symptoms such as snoring and excessive daytime sleepiness (EDS). EDS is a common symptom among hypertensive patients. This interesting and remarkable systematic review aims to comprehensively survey the current literature on the prevalence and association of EDS among hypertensive patients. PubMed, SCOPUS, Web of Science, and Science Direct were systematically searched for relevant literature. Rayyan QRCI (Rayyan Systems Inc., Cambridge, MA) was employed throughout this comprehensive process. This review included nine studies with a total of 58,517 patients, and 34,398 (58.8%) were males. EDS prevalence among hypertensive patients ranged from 12.1% to 88.3%. This review stated that individuals with hypertension (HTN) had EDS that was worse than that of patients with normotension. In one of the studies included in this analysis, sympathovagal imbalance was noted alongside EDS and HTN. Older age, primary education, being overweight, working, obesity, depression, and having had the condition for longer are all associated with EDS in HTN patients.

## Introduction and background

Cardiovascular morbidity and mortality are significantly impacted by hypertension (HTN), which is a key risk factor for cardiovascular disease, including stroke [1-3]. HTN is considered high intravascular pressure, impairing circadian blood pressure (BP). In addition, it has been shown to contribute to poor clinical outcomes, potentially [4-6]. According to Mills et al. [7], it affected 1.39 billion people worldwide in 2010 (31% of all adults), a rise in prevalence of 5.2% from 2000 to 2017.

BP exhibits a consistent circadian rhythm characterized by a decrease of 10%-20% during nighttime compared to daytime, primarily due to inherent neuroendocrine oscillations and various contributing factors [5]. People with HTN had lower-quality sleep than individuals with normal BP, according to earlier research [8-10]. Additionally, it has been demonstrated that sleep issues are linked to greater rates of all-cause mortality [11], as well as an increased risk of HTN [10,12], vascular inflammation [13], and uncontrolled and treatment-resistant HTN [14]. According to the findings of a study by Li et al. [15], treating sleep disturbances in hypertensive patients can lower BP.

About one-third of the population experiences sleep difficulties, such as insomnia or EDS, which makes them a serious therapeutic issue. The most common definition of insomnia is the existence of a subjective report of trouble falling asleep, which results in insufficient or poor-quality sleep [16].

EDS is a prevalent clinical issue. It is among the most significant effects of sleep disorders and is linked to a lower quality of life, traffic accidents, and workplace mishaps [17]. EDS is characterized by an almost daily inability to maintain vigilance and alertness for the majority of the day when the individual is expected to be awake, with sleep occurring accidentally or at the wrong times. According to estimates [18-21], it affects between 10% and 20% of the general population and up to 68% of certain patient populations [21]. Numerous studies, particularly those involving people who had sleep apnea syndrome (SAS), revealed an independent relationship between EDS and HTN [21,22]. This systematic review aims to comprehensively investigate the recent literature on the prevalence and association of EDS among hypertensive patients.

## Review

Methodology

This systematic review complied with established criteria (Preferred Reporting Items for Systematic Reviews and Meta-Analyses, PRISMA) [23].

Study Design and Duration

This systematic review was conducted in September 2023.

Search Strategy

A thorough search was conducted across four main databases, including PubMed, SCOPUS, Web of Science, and Science Direct, to locate the pertinent literature. We limited our search to English and considered each database's particular needs. The studies corresponding to the following keywords were located using PubMed Mesh terms; "Excessive daytime sleepiness," "Sleep disturbances," and "Hypertension." The Boolean operators "OR" and "AND" matched the required keywords. Publications with full English text, available free articles, and human trials were among the search results.

Selection Criteria

We considered the following criteria for inclusion in this review: studies that investigated the recent literature on the prevalence and association of EDS among patients with arterial HTN; studies that included adults and adolescents; studies conducted within the last five years (2019-2023) on human subjects; studies in the English language; and free accessible articles.

Data Extraction

Rayyan QCRI (Rayyan Systems Inc., Cambridge, MA) was used to check the output of the search technique for duplication [24]. The researchers assessed the titles' and abstract relevance by altering the combined search results with the inclusion/exclusion criteria. The reviewers thoroughly scrutinized each paper that matched the inclusion criteria. The writers discussed dispute-resolution approaches. The authorized study was uploaded using a previously generated data extraction form. The authors extracted data about the study titles, authors, study year, country, participants, gender, objectives, prevalence of EDS, and main outcomes. A separate sheet was created for the risk of bias assessment.

Strategy for Data Synthesis

Summary tables were created using data from relevant studies to provide a qualitative interpretation of the findings and study components. After retrieving the data for the systematic review, the most efficient strategy to use the data from the included study articles was chosen.

Risk of Bias Assessment

The quality of the included studies was assessed using the ROBINS-I risk of bias assessment approach for non-randomized trials of treatments [25]. Confounding, participant selection for the study, classification of interventions, deviations from intended interventions, missing data, assessment of outcomes, and selection of the reported result were the seven themes evaluated.

Results

Search Results

**Figure 1:**
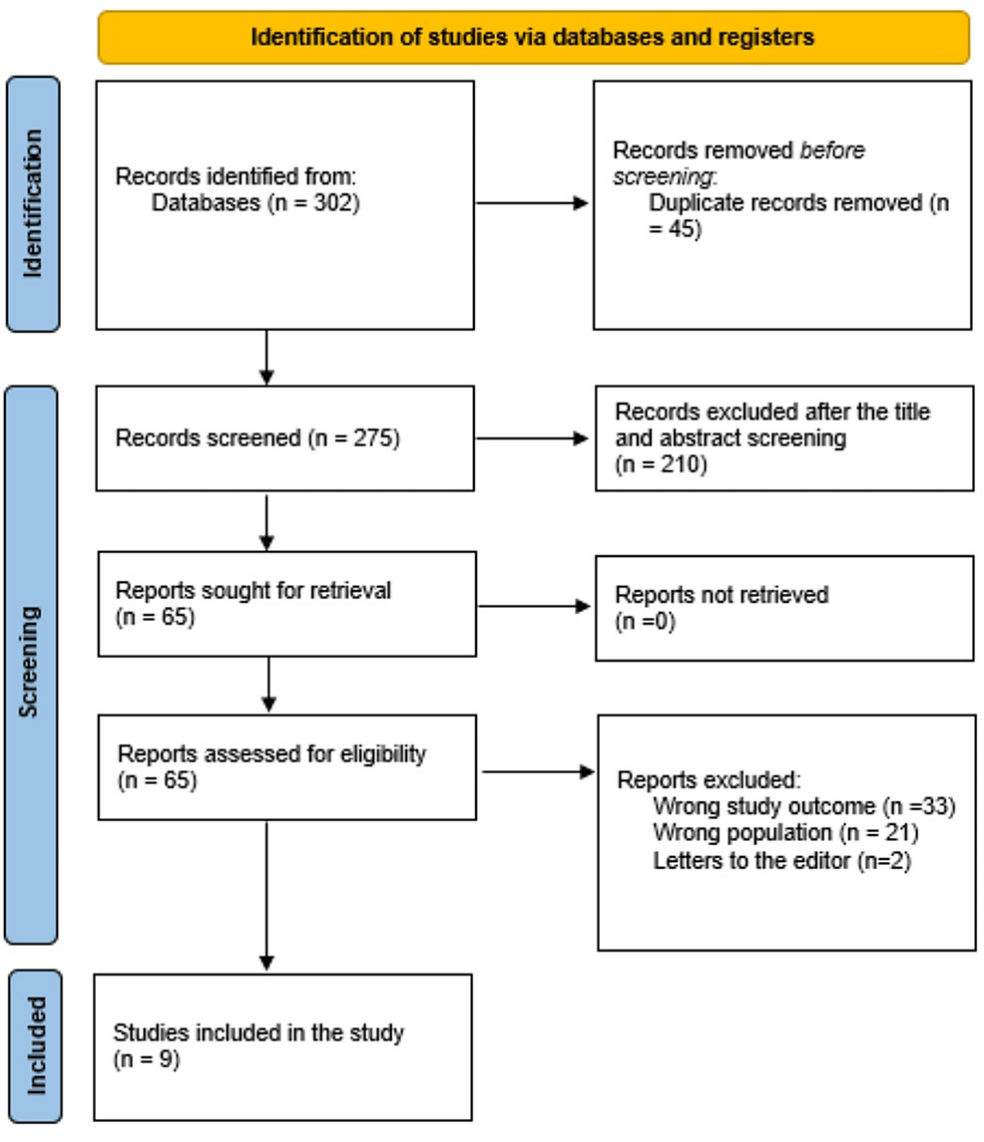
PRISMA flowchart summarizes the study selection process PRISMA: Preferred Reporting Items for Systematic Reviews and Meta-Analyses

A total of 302 study articles resulted from the systematic search, and 45 duplicates were deleted. Title and abstract screening were conducted on 275 studies, and 210 studies were excluded. A total of 65 reports were sought for retrieval, and no articles were retrieved. Finally, 65 studies were screened for full-text assessment, 33 were excluded for wrong study outcomes and 21 for the wrong population type, and two articles were letters to the editors. Nine eligible study articles were included in this systematic review. A summary of the study selection process is presented in Figure 1.

Characteristics of the Included Studies

Table 1 presents the sociodemographic characteristics of the included study articles. Our results included nine studies, with a total of 58,517 patients, and 34,398 (58.8%) were males. Three articles were cross-sectional studies, two were case controls, two were retrospective in nature, one was a randomized control trial (RCT), and one was prospective in nature.

**Table 1:** Sociodemographic characteristics of the included participants SD: Standard deviation * Minimum and maximum age are presented.

Study	Study design	Country	Participants	Mean ± SD age (years)	Males (%)
Uchmanowicz et al., 2019 [26]	Cross-sectional	Poland	100	65.5 ± 15.6	54 (54)
Tam et al., 2019 [27]	RCT	China	280	51.7 ± 11.9	204 (72.9)
Kaya et al., 2020 [28]	Case-control	Turkey	266	54.9 ± 9.1	204 (76.7)
Meng et al., 2021 [29]	Case-control	China	161	23-68*	136 (84.4)
Lee et al., 2019 [30]	Retrospective cohort	Korea	4954	50.8 ± 8.5	2338 (47.8)
Bouloukaki et al., 2020 [31]	Prospective cohort	Multi-centered	4,732	52.4 ± 12.5	2,579 (55)
Koo et al., 2022 [32]	Cross-sectional	USA	5052	55.3 ± 12.8	1866 (36.9)
Pefura-Yone et al., 2020 [33]	Cross-sectional	Cameroon	8288	27-54*	3709 (44.8)
Ulander et al., 2022 [34]	Retrospective cohort	Sweden	34684	55.7 ± 13.7	23308 (67.2)

Table 2 presents the clinical characteristics. EDS prevalence among hypertensive patients ranged from 12.1% [30,33] to 88.3% [28]. All of the included studies reported that EDS was worse among hypertensive than normotensive patients. Older age, primary education, being overweight, working, obesity, depression, and having had the condition for longer are all associated with EDS in HTN patients [26,33,34]. Meng et al. reported that sympathovagal imbalance was seen along with EDS and HTN [29].

**Table 2:** Clinical characteristics and outcomes of the included studies NM: Not mentioned

Study	Objectives	Prevalence of EDS	Main outcomes	ROBIN-I
Uchmanowicz et al., 2019 [26]	To assess how insomnia and EDS affect older HTN patients' quality of life (QOL).	39 (39)	Sleep issues significantly lower QOL in HTN patients, particularly in the physical category of the QOL questionnaire. Older age, primary education, being overweight, working, and having had the condition for longer are all associated with EDS and insomnia in HTN patients.	High
Tam et al., 2019 [27]	To see if there are any noticeable variations in EDS between hypertensive participants who have moderate to severe OSA and normal subjects.	NM	Subjective EDS was considerably worse in hypertensive patients compared to normotensive subjects with moderate to severe OSA.	NA
Kaya et al., 2020 [28]	To identify the OSA clinical and polysomnographic characteristics that are significantly related to HTN.	235 (88.3)	Compared to the normotensive group, the HTN group's sleep efficiency was considerably worse.	Moderate
Meng et al., 2021 [29]	To determine whether patients with OSA shared any similar pathophysiologic factors that contributed to their reported EDS and HTN.	NM	A more severe form of the respiratory condition is OSA, accompanied by excessive EDS and HTN. Sympathovagal imbalance was seen along with EDS and HTN, and the co-occurrence of these two disorders may be connected to lower plasma ACh levels.	High
Lee et al., 2019 [30]	To determine if age and gender affect the relationship between frequent snoring and HTN.	283 (12.1%)	Only in men aged 45 years was frequent snoring substantially related to a 1.5 times increased risk for incident HTN than never snoring after controlling for known cardiovascular risk factors. Regardless of EDS frequent snoring was strongly linked to an increased risk for the development of HTN in this age range.	Moderate
Bouloukaki et al., 2020 [31]	In the European Sleep Apnea Database cohort, look at the relationship between mild OSA and systemic arterial HTN.	2129 (47)	AHI levels of less than 11 occurrences per hour were strongly linked to an increased risk of developing HTN. Despite the lack of additional daytime or sleep-related symptoms, this may result in the early identification of people at risk and treatment.	Moderate
Koo et al., 2022 [32]	To find out if the risk factors for OSA (such as obesity, loud snoring, and EDS) differ in terms of aldosterone status from the severity and management of HTN.	2,480 (49.1)	In adults of African American descent, risks for sleep disruptions were positively correlated with resistant HTN and higher levels of aldosterone.	Moderate
Pefura-Yone et al., 2020 [33]	To examine the relationship between EDS and high blood pressure and to identify the causes of EDS in hypertensive people.	1000 (12.1)	An independent link between EDS and HTN was not discovered. With HTN, obesity is linked to EDS.	Moderate
Ulander et al., 2022 [34]	To pinpoint factors that contribute to EDS in the SESAR cohort of patients with newly diagnosed OSA.	14740 (42.5)	Higher ESS scores were linked to depression, while lower levels were linked to atrial fibrillation and HTN. Compared to the apnea-hypopnea index, the oxygen desaturation index better predicted EDS.	Moderate

Discussion

This systematic review reported that the prevalence of EDS among hypertensive patients ranged from 12.1% [30,33] to 88.3% [28]. There are few reports related to EDS and HTN. When used on patients with obstructive sleep apnea, Feng et al.'s research suggests that the Epworth Sleepiness Scale may serve as a predictor of BP profile [35]. Additionally, it has been shown in the past that, in contrast to patients without EDS HTN, BP in obstructive sleep apnea (OSA) patients with EDS reduced after CPAP therapy [36].

All of the included studies in this review reported that EDS was worse among hypertensive than normotensive patients [26-34]. This study suggests that subjective ratings of EDS are also linked to BP and HTN. Sleep disorders, such as obstructive sleep apnea, have been linked to BP increases and HTN [37], as well as to EDS. The correlation between EDS and BP is a significant discovery and supports earlier hypotheses that EDS can be used to identify older people at higher risk for cardiovascular problems [38]. It also emphasizes the importance of researching whether diagnosing and treating EDS can help lower BP and, eventually, reduce morbidity and death from cardiovascular diseases.

One study in this review reported that sympathovagal imbalance was seen, along with EDS and HTN [29]. The following is a proposed mechanism for how hypoxia raises BP. Chemoreceptors and baroreceptors in the carotid artery control sympathetic nerve activity. A fall in arterial blood oxygen levels activates the carotid body, which in turn stimulates the sympathetic center in the brain, stimulating the afferent nerve and causing the efferent sympathetic nervous system to become hyperactive. An elevation in BP is made possible by increased sympathetic nerve activity and a weaker baroreflex. Due to functional alterations in chemoreceptors and baroreceptors, chronic severe OSA will raise BP both during the day and at night, in addition to the transitory increase that occurs at night [39].

We found that older age, primary education, being overweight, working, obesity, depression, and having had the condition for longer are all associated with EDS in HTN patients [26,33,34]. Earlier claims that EDS can be used to identify older people who are more likely to experience cardiovascular diseases. Patients with OSA who are obese should strive to lose weight with a program that includes food counseling and exercise.

Patients' sleep habits and behavior should be evaluated, and care should be tailored to these findings. It may be necessary for a patient and/or important as part of nursing care to assess sleep patterns and practical techniques that may enhance patients' sleep. The medical staff should suggest a drug schedule in the event of persistent sleep issues. In elderly people, EDS is a prevalent but usually misinterpreted symptom. In the Cardiovascular Health Study, 20% of participants (aged 65 or older) reported feeling drowsy during the day [40]. EDS, despite commonly thought of as a benign condition, has been linked to a higher risk of cardiovascular death [41]. Additionally, although EDS is common in older populations, it may have less to do with age and be more related to the elderly's health issues [42].

There are numerous subclinical cases of OSA, even though the patient or family members typically become aware of it through symptoms such as snoring and EDS. In OSA, nighttime hypoxia and arousal frequently increase sympathetic nerve activity and raise BP during the night or early in the morning. When OSA is severe, sympathetic hyperactivity lasts all day, which causes a persistent rise in daytime BP. Atherosclerosis would eventually cause the development of cardiac and cerebrovascular illnesses in addition to HTN, and it is also known that impairment of glucose metabolism results from increased sympathetic nerve hyperactivity [11]. Actually, due to the development of comorbidities and, in particular, when oxygen saturation is decreased by sleep apnea to below 78%, and even the risk of sudden death being greatly elevated [13], it has been observed that the prognosis of severe OSA is particularly dismal [12].

One of the limitations is the scarcity of studies on the processes, and another is the high prevalence of obesity and OSA, which can have an impact on the micro- and macro-structures of sleep.

## Conclusions

This review stated that individuals with HTN had EDS that was worse than that of patients with normotension. In one of the studies included in this analysis, sympathovagal imbalance was noted alongside EDS and HTN. Older age, primary education, being overweight, working, obesity, depression, and having had the condition for longer are all associated with EDS in HTN patients.
